# Adenosine-5′-phosphosulfate – a multifaceted modulator of bifunctional 3′-phospho-adenosine-5′-phosphosulfate synthases and related enzymes

**DOI:** 10.1111/febs.12252

**Published:** 2013-04-17

**Authors:** Jonathan W Mueller, Naeem Shafqat

**Affiliations:** Centre for Endocrinology, Diabetes, and Metabolism (CEDAM), School of Clinical and Experimental Medicine, University of Birmingham, Institute of Biomedical ResearchUK

**Keywords:** 3′-phospho-adenosine-5′-phosphosulfate (PAPS) synthase, adenosine-5′-phosphosulfate (APS), enzyme inhibitor, protein stability, sulfation/sulfonation/sulfurylation

## Abstract

All sulfation reactions rely on active sulfate in the form of 3′-phospho-adenosine-5′-phosphosulfate (PAPS). In fungi, bacteria, and plants, the enzymes responsible for PAPS synthesis, ATP sulfurylase and adenosine-5′-phosphosulfate (APS) kinase, reside on separate polypeptide chains. In metazoans, however, bifunctional PAPS synthases catalyze the consecutive steps of sulfate activation by converting sulfate to PAPS via the intermediate APS. This intricate molecule and the related nucleotides PAPS and 3′-phospho-adenosine-5′-phosphate modulate the function of various enzymes from sulfation pathways, and these effects are summarized in this review. On the ATP sulfurylase domain that initially produces APS from sulfate and ATP, APS acts as a potent product inhibitor, being competitive with both ATP and sulfate. For the APS kinase domain that phosphorylates APS to PAPS, APS is an uncompetitive substrate inhibitor that can bind both at the ATP/ADP-binding site and the PAPS/APS-binding site. For human PAPS synthase 1, the steady-state concentration of APS has been modelled to be 1.6 μm, but this may increase up to 60 μm under conditions of sulfate excess. It is noteworthy that the APS concentration for maximal APS kinase activity is 15 μm. Finally, we recognized APS as a highly specific stabilizer of bifunctional PAPS synthases. APS most likely stabilizes the APS kinase part of these proteins by forming a dead-end enzyme–ADP–APS complex at APS concentrations between 0.5 and 5 μm; at higher concentrations, APS may bind to the catalytic centers of ATP sulfurylase. Based on the assumption that cellular concentrations of APS fluctuate within this range, APS can therefore be regarded as a key modulator of PAPS synthase functions.

## Introduction

Sulfated biomolecules are omnipresent in biology. In vertebrates, bone and cartilage development only functions properly with chondroitin and heparan sulfates 1, and phase II biotransformation of xenobiotics and drugs includes sulfation as a key mechanism 2. The tissue-specific and systemic activation of steroid hormones is regulated by sulfation 3, major protein components of the blood clotting cascade are sulfated, and human immunodeficiency virus (HIV) needs a sulfated coreceptor, CXCR5, to invade its host cells 4. In plants, glucosinolates represent a major class of sulfated secondary metabolites that mediate interactions with herbivores and pathogens 5. In all of these areas, highly diverse biomolecules are modified by the attachment of a sulfate group, a central process called sulfation (also termed sulfonation or sulfurylation). In humans, > 50 sulfotransferase enzymes are responsible for various sulfation processes 6, and they can be found in the cellular nucleus 7, the cytoplasm 8, and the Golgi apparatus 9. All eukaryotic sulfotransferases depend on the provision of active sulfate in the form of 3′-phospho-adenosine-5′-phosphosulfate (PAPS) for their proper action. Hence, the importance of PAPS for sulfation can rival that of ATP for phosphorylation processes.

**Fig. 1:**
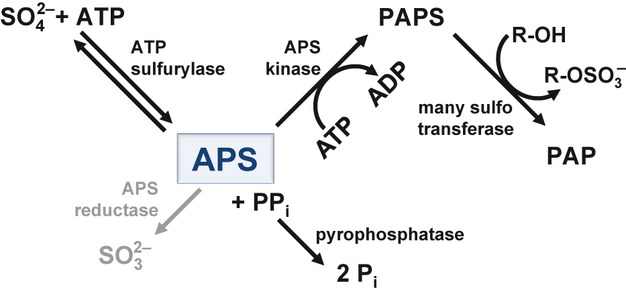
Schematic of sulfate metabolism. Sulfate is converted to the sulfonucleotide APS (boxed and highlighted in blue) by the ubiquitous ATP sulfurylase. APS represents a metabolic branchpoint in bacteria and plants, where it is reduced by APS reductase (highlighted in grey) to sulfite, and finally incorporated into primary metabolites after further reduction. Alternatively, APS is phosphorylated by APS kinase to the universal sulfate donor PAPS. In metazoans, ATP sulfurylase and APS kinase reside on one polypeptide, the bifunctional PAPS synthase.

Sulfate needs to be activated, because this highly stable oxyanion normally resists gentle attempts to make it undergo chemical bonding. The activation system is a multistep process (Fig. 1). First, ATP sulfurylase transfers the AMP moiety of ATP onto sulfate, with concomitant release of pyrophosphate (PP_i_); the product is adenosine-5′-phosphosulfate (APS). This reaction is highly endergonic, so equilibrium resides mostly at the educt side. It is of interest that the reverse reaction – starting with APS and PP_i_, and producing ATP – is a key feature utilized by next-generation sequencing techniques 10. To thermodynamically draw the reaction to completion, subsequent steps are needed for sulfate activation: (a) cleavage of PP_i_ by ubiquitous pyrophosphatases; (b) an additional phosphorylation of the ribose ring catalysed by APS kinase; and/or (c) reduction of APS or PAPS to sulfite and further to sulfide-containing metabolites.

The sulfonucleotide APS fulfils multiple roles in this process. It is the product of the sulfurylase reaction, and is also a potent product inhibitor of sulfurylase enzymes in many organisms. In auxotrophic plants and bacteria, the phosphorylation of APS to PAPS is not obligatory for reductive sulfate assimilation, as the exergonic conversion of APS to sulfite by APS reductase is most probably sufficient to pull the ATP sulfurylase reaction to completion. Hence, APS represents a metabolic branchpoint for sulfate metabolism in plants 5. When APS reduction is not an option, APS kinase becomes important to pull the sulfurylase reaction by phosphorylation of the ribose 3′-OH group, finally yielding PAPS. Generally, APS kinases are also inhibited by APS. Finally, APS has recently been recognized as a specific stabilizer of the human PAPS synthases, PAPS synthase 1 (PAPSS1) and PAPS synthase 2 (PAPSS2) 11, bifunctional enzymes that consist of ATP sulfurylase and APS kinase domains connected by a flexible linker. This review highlights the various regulatory roles of APS in the overall process of PAPS biosynthesis. On the basis of considerations about the inhibition of APS kinase and ATP sulfurylase enzymes, we suggest how PAPS synthase stabilization by APS may occur mechanistically. Furthermore, the role of the related nucleotides, PAPS and 3′-phospho-adenosine-5′-phosphate (PAP), in the regulation of sulfation pathways is also briefly reviewed.

## APS as an inhibitor of the ATP sulfurylase

ATP sulfurylases are ubiquitous enzymes that catalyze the first step of biochemical sulfate activation, the reaction of inorganic sulfate with ATP to form APS and PP_i_. The crucial mechanistic feature of this reaction is an ATP-binding mode that is unique to ATP sulfurylases and different from that of related nucleotidylyl transferases. At the sulfurylase, ATP presumably adopts an unusual U-shaped conformation to allow the in-line attack of sulfate on the α-phosphorus. This binding mode was suggested on the basis of an enzyme–APS–PP_i_ complex of yeast ATP sulfurylase, on which an enzyme–ATP complex was modelled, as an ATP complex is not available for any sulfurylase 12.

**Fig. 2:**
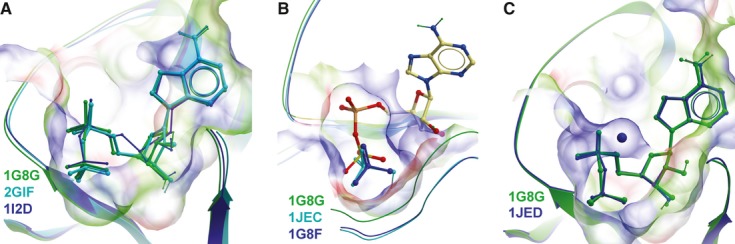
Ligand binding in ATP sulfurylase structures. (A) Three different APS complexes of sulfurylases, from yeast (Protein Data Bank ID 1G8G, green) 12, human PAPS1 (2GIF, cyan) (unpublished), and the fungus *P. chrysogenum* (1I2D, blue) 18, were overlaid. In all structures, the APS sulfonucleotide is bound in a very similar manner. (B) The APS complex of yeast sulfurylases (1G8G, green) is overlaid with two structures of yeast sulfurylase complexed to thiosulfate (1JED, cyan) 19 and sulfate (1G8F, blue) 12. The APS complex structure is more compact than the other two structures; however, the positioning of the APS sulfate is very similar to that of sulfate and thiosulfate. (C) The binding mode of APS (1G8G, green) is compared with that of an ADP–sulfurylase complex (1JED, blue). The two nucleotides are bound in a nearly identical manner; however, the ADP complex additionally contains a bound magnesium ion.

ATP sulfurylase enzymes from yeast, fungi and humans show pronounced product inhibition by APS 13–15. Considering the extremely small equilibrium constant for the APS synthesis direction 16, a very high affinity for the physiological reaction product of the sulfurylase reaction may be compensatory. APS is competitive with both substrates, ATP and sulfate 15, indicating direct binding to the catalytic center, which can be visualized in an overlay of several sulfurylase–APS complex structures (Fig. 2A). In all of these structures, APS is bound in a conformation that is distinctively different from the presumed ATP-binding mode described above. We compared the binding mode of APS at the sulfurylase with sulfurylase structures containing sulfate or thiosulfate (Fig. 2B), there it became apparent that the phosphosulfate precisely overlayed the bound sulfate group. However, the APS complex is more compact than the other structures. In addition, the mode of APS binding was compared with that of an ADP–sulfurylase complex (Fig. 2C). Unlike ATP, the ADP molecule is bound in a similar conformation as APS. It is noteworthy that a magnesium ion is complexed to ADP that is absent in all available APS–sulfurylase complex structures. This magnesium ion may be a decisive feature in discriminating between APS and ADP nucleotides (see below). However, it has been suggested that the lack of one negative charge at the β-position (sulfate instead of phosphate) plays a major role in the enzymatic discrimination between these two nucleotides 17.

## The APS dead-end complex of APS kinases

APS kinase is responsible for the phosphorylation of APS at the ribose 3′-OH group, and the structures of APS kinases from a variety of species are very similar. Kinetic studies with the enzyme from *Penicillium chrysogenum* have established an ordered reaction mechanism, whereby MgATP binds first, followed by the second substrate APS 20. After catalysis, PAPS leaves first, followed by ADP. Other studies on the APS kinase, however, suggested that ATP and APS bind to the enzyme in random order 21.

**Fig. 3:**
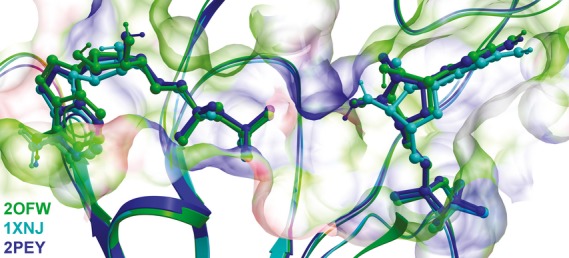
APS dead-end complexes of APS kinase. Three structures of APS kinase of human PAPSS1 are overlaid, one complexed to two APS molecules (2OFW, green) 27, one containing an ADP molecule and an APS molecule (1XNJ, cyan) 29, and one crystallized with a desoxy-ADP (dADP) molecule and an APS molecule (2PEY, blue) 28. The binding pocket depicted on the left represents the ADP/ATP-binding site. This pocket is occupied by APS, ADP and dADP, respectively, in the three structures. The binding pocket on the right is the APS/PAPS-binding site, which is occupied by an APS molecule in all three structures. The structural representations in Figs 2 and 3 were created with molsoft icm molbrowser 3.7.

A hallmark of both monofunctional APS kinases 21–24 and of APS kinase from bifunctional human PAPS synthase 13 is their pronounced substrate inhibition by APS. This inhibition is uncompetitive in nature with respect to ATP, suggesting the formation of the dead-end enzyme–ADP–APS complex after completion of the enzymatic reaction, when PAPS leaves the active site and APS rebinds to the same site before ADP dissociation. The existence of an enzyme–ADP–APS complex is supported by the highly increased affinity of APS for an enzyme–ADP complex relative to the apoenzyme 25,26. A symmetrical enzyme–APS–APS complex of the dimeric APS kinase domain of human PAPSS1 may be taken as a model for this dead end in the catalytic pathway 27 (Fig. 3). Truncated and point mutants have been reported that lack substrate inhibition 28, resulting in an asymmetric dimer structure, similar to the conformation of APS kinase within the context of the full-length enzyme 29. Sekulic *et al*. concluded that this asymmetry helped in reducing the substrate inhibition of APS 2007. Recently reported heterodimers of PAPSS1 and PAPSS2 – dimeric complexes with inbuilt asymmetry – did indeed show slightly diminished APS inhibition 30, with an APS concentration for maximal APS kinase activity of 15 μm APS. This value was in agreement with previous measurements 13. When one subunit of dimeric APS kinases is considered at a time, all three available dead-end complexes show exactly the same mode of nucleotide binding (Fig. 3).

## Bifunctional PAPS synthases are stabilized by APS

Within the cell, strong product inhibition of the sulfurylase enzyme by APS would not be an obstacle if the next enzyme in the sulfate activation sequence (APS kinase) had a high affinity for APS. All vertebrates, and at least all sequenced invertebrates, including insects and worms, have bifunctional PAPS synthases in the form of a C-terminal ATP sulfurylase domain and an N-terminal APS kinase domain connected by a short irregular linker 11. Intermediate channeling has been suggested for this type of PAPS synthase, as a possible way to circumvent APS inhibition 31, but this has been clearly disproved biochemically 13 and structurally 29 for human PAPSS1. Channeling of the intermediate APS has unambiguously been shown only for the differentially arranged ATP sulfurylase/APS kinase resulting from gene fusions from some bacteria with an N-terminal sulfurylase and a C-terminal APS kinase domain 16. A third type of sulfate-activating enzyme has been described, involving GTPase activity 16; these two classes of enzymes are, however, beyond the scope of this article.

For human PAPS synthases, the maximal attainable APS kinase activity of recombinant PAPSS1 is clearly lower than the maximal attainable activity of the ATP sulfurylase under the same conditions 13. Because of this activity imbalance, PAPS synthesis rates within the cell will depend on an unknown and ever-changing concentration of the accumulating (and inhibitory) APS intermediate. Lansdon *et al*. 2004 have calculated an approximate steady-state APS concentration of 1.6 μm at the PAPSS1 enzyme, which can increase up to 60 μm under conditions of sulfate excess.

**Fig. 4:**
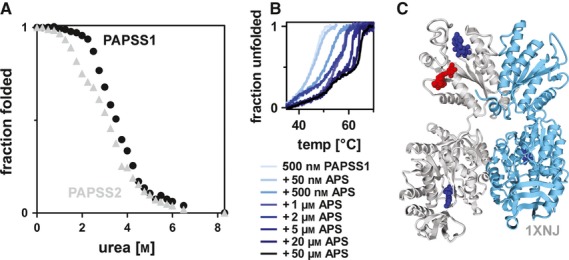
Different stabilities of human PAPS synthases and specific stabilization by APS. (A) Unfolding of human PAPS synthases at increasing concentrations of urea in the absence of APS. To reach the midpoint of unfolding requires 400 mm less urea for PAPSS2 than for PAPSS1. (B) Thermal unfolding of human PAPSS1 in the presence of various APS concentrations. Unfolding was monitored by intrinsic tryptophan fluorescence and CD spectroscopy in (A) and (B), respectively. (C) An APS complex of full-length PAPSS1 has been previously been reported (1XNJ) 29, and may represent the mechanism by which APS stabilizes PAPS synthases. The two subunits of the dimeric protein are shown in grey and light blue, the ADP molecule bound to only one of the two APS kinase domains is shown in red, and the three APS molecules are shown in blue. (A) and (B) were originally published in the *Journal of Biological Chemistry* 11. © the American Society for Biochemistry and Molecular Biology.

Recently, we made the unexpected observation that the two human isoforms of PAPS synthase differ remarkably in their stability towards chemically and thermally induced unfolding 11. In a search for stabilizing substances for the PAPS synthases, we tested the substrates, intermediates and products of PAPS formation itself for their ability to stabilize these fragile proteins. Sulfate and AMP had no effect on thermal unfolding transitions of PAPS synthases; ATP had a slight stabilizing effect on PAPSS2, and moderate effects on PAPSS1 and PAPSS2 stability were observed for ADP and PAPS. The sulfonucleotide APS had the most prominent effect on the unfolding of PAPS synthases (Fig. 4). At equimolar concentrations, APS already had a remarkable effect on the stability of PAPSS1, increasing the midpoint of the unfolding transition by 4 °C. APS at 50 μm even caused changes in the overall form of the unfolding transitions, as it stabilized an unfolding intermediate by > 16 °C in both PAPS synthases. From the analysis of several point mutants, the transition stabilization at higher APS concentrations was attributed to the unfolding of the sulfurylase domain of bifunctional PAPS synthases 11.

This dual stabilizing effect can now be interpreted in the light of the above-described inhibition mechanisms. First, the high-affinity enzyme–ADP–APS complex forms within the APS kinase domain. Subsequently, the catalytic centers of ATP sulfurylase are filled with the competitive inhibitor APS. Such an APS-stabilized full-length PAPS synthase is possibly best visualized by reference to the previously reported APS complex of PAPSS1 (Fig. 4C) 29. In that study, the recombinant protein was found to carry a tightly bound ADP molecule that did not separate during the purification procedure; this then allowed for the formation of an enzyme–ADP–APS_3_ complex upon soaking of the crystals in 1 mm APS 29. If this complex corresponds to the stabilized protein species from our measurements, we also need to have had ADP in our protein preparations, an assumption that we recently confirmed with ion-pairing RP-HPLC analysis, using a C18 column run in isocratic mode in 50 mm potassium phosphate (pH 6.6), 10 mm tetrabutylammonium bromide, and 8% acetonitrile, according to 32. The ADP/protein ratios were found to be 0.47 ± 0.13 and 0.50 ± 0.10, averaged over three different batches of PAPSS1 and PAPSS2, respectively, and were in reasonably good agreement with one ADP molecule being bound per protein dimer. For APS kinase 1 of *Arabidopsis thaliana*, affinity values for different nucleotides were recently reported 25 that seem to support our model. The apoenzyme weakly bound APS (*K*_d_ = 66.7 ± 10.5 μm); the affinity increased significantly when the enzyme was preloaded with ADP (3.3 ± 0.70 μm), and even further in the presence of magnesium (0.60 ± 0.20 μm) 25.

## Chemical considerations regarding phosphosulfate-containing nucleotides

What is so special about the sulfonucleotide APS? The chemical hallmark of sulfate activation is the phosphoric–sulfuric acid anhydride bond in APS, with a Δ*G*_0_′ for hydrolysis in the range of −19 kcal·mol^−1^ 33. However, this bond is also present in PAPS, and both compounds have been considered to be ‘unstable’. The hydrolysis of phosphosulfate-containing model compounds has been studied previously. This bond was recognized to be highly labile to acidic hydrolysis, but to be stable with half-lives of several days at pH values > 6.5 34,35; this also applied to various other phosphosulfate-containing nucleotides (J. Jemielity, personal communication). Moreover, hydrolysis of the phosphosulfate bond was facilitated in the presence of divalent metal ions 35,36; hence, buffered stock solutions of these compounds should also contain chelating agents such as EDTA 37. Accordingly, solutions at pH 8 should be stable when frozen, and, at refrigerator temperatures, hydrolyze at a rate of < 1% per day.

The sulfonucleotides APS and PAPS have recently been synthesized chemically 37, and no differences in stability between the two compounds were observed (J. Jemielity, personal communication). This is interesting, because PAPS and APS are commercially available in strikingly different grades of purity. PAPS lithium salt (A1651; Sigma, Taufkirchen, Germany) is available at only 60% purity, and the purity of APS sodium salt (A5508; Sigma), at ∼ 90%, is below the value normally found for other chemicals. The most likely impurities are AMP and PAP, the nucleotides remaining after the hydrolysis of the phosphosulfate moiety. However, if and why the two compounds should differ in their stability remains currently unclear.

## Other nucleotides are also effectors for sulfation pathways

### PAPS

Sulfation pathways also involve other nucleotides that, in return, also profoundly modulate sulfate-activating enzymes. The ATP sulfurylase enzyme from *P. chrysogenum* has an extended C-terminal domain that is very similar to APS kinases, but is devoid of APS kinase activity. This domain confers allosteric regulation to this ATP sulfurylase by PAPS binding 38. Other ATP sulfurylases also contain domains similar to APS kinases, and vice versa 39; these may be remnants of ancient bifunctional enzymes adapted for other purposes. Interestingly, Kopriva *et al*. also found increased transcript levels for genes of glucosinolate biosynthesis in *Arabidopsis* when bacterial APS kinase was overexpressed 5. Glucosinolates constitute a major class of sulfated secondary metabolites in plants, and their biosynthesis requires PAPS as the sulfate donor. From their findings, they concluded that PAPS or its derivatives may be involved in cellular signalling in plants 5. In analogy to this, negative feedback regulation might also play a role in mammalian cells to elicit a transcriptional response upon low APS levels, to increase overall PAPS synthesis.

### PAP

Yet another nucleotide may be important for a functional understanding of PAPS synthases: PAP. This is the residual product when a sulfotransferase uses PAPS for biological sulfation. Several studies have reported regulatory roles for PAP in drought and high-light signalling in *Arabidopsis* 40, or more generally for stress-responsive gene expression in plants 41. Moreover, high PAP concentrations were reported to be toxic in bacteria, owing to inhibition of RNA 5′–3′-exonucleases 42 and PAPS reductases. However, PAP does not directly have a strong effect on human PAPS synthase stability 11. PAP may be subject to degradation by various bisphosphatases, another emerging field that has been reviewed recently 43.

## Future directions

PAPS synthases seem to actively shuttle between the nucleus and cytoplasm 44, and it remains to be seen whether high or low cellular APS levels influence this localization pattern. Stabilization and inhibition by the sulfonucleotide APS is surely not the only way in which PAPS synthase function is modulated. Within the cell, PAPS synthases may also associate with chaperones or other stabilizing proteins. The first attempts to find such proteins by yeast two-hybrid screens did not result in plausible interaction partners 45. However, on the basis of triple fusion proteins of APS kinase, ATP sulfurylase and pyrophosphatase within the genomes of the fully sequenced marine diatoms *Thalassiosira pseudonana* and *Phaeodactylum tricornutum* 46, a functional interaction with pyrophosphatase has been suggested 47. Such triple gene fusion in algae may be especially interesting, as diatoms and haptophytes reduce APS and not PAPS, so the APS kinase reaction of this complex seems to be not actually required in this case.

APS and the related nucleotides PAPS and PAP modulate the functions of various enzymes from sulfation pathways. For bifunctional PAPS synthases, which are at the heart of mammalian sulfation reactions, APS is not only an intermediate of overall PAPS production, but also serves as an inhibitor of the sulfurylase and the APS kinase activities, and as a stabilizer for the fragile PAPS synthases. Changing intracellular concentrations of the sulfonucleotide APS (and putatively also of other nucleotides involved) may have a tremendous influence on cellular sulfation processes. However, intracellular concentrations for these metabolites have not yet been quantified.

## Abbreviations

APS: adenosine-5′-phosphosulfate
PAP: 3′-phospho-adenosine-5′-phosphate
PAPS: 3′-phospho-adenosine-5′-phosphosulfate
PAPSS1: 3′-phospho-adenosine-5′-phosphosulfate synthase 1
PAPSS2: 3′-phospho-adenosine-5′-phosphosulfate synthase 2
PP_i_: pyrophosphate
